# Internalization of Polymeric Bacterial Peptidoglycan Occurs through Either Actin or Dynamin Dependent Pathways

**DOI:** 10.3390/microorganisms10030552

**Published:** 2022-03-03

**Authors:** Narcis I. Popescu, Jackie Cochran, Elizabeth Duggan, Jędrzej Kluza, Robert Silasi, Kenneth Mark Coggeshall

**Affiliations:** 1 Department of Arthritis and Clinical Immunology, Oklahoma Medical Research Foundation, Oklahoma City, OK 73104, USA; jackie-cochran@omrf.org (J.C.); elizabeth-duggan@omrf.org (E.D.); jedrzej-kluza@omrf.org (J.K.); 2 Cardiovascular Biology Research Program, Oklahoma Medical Research Foundation, Oklahoma City, OK 73104, USA; robert-silasimansat@omrf.org

**Keywords:** peptidoglycan, bacteria, monocytes, internalization, CIE

## Abstract

Peptidoglycan (PGN), a polymeric glycan macromolecule, is a major constituent of the bacterial cell wall and a conserved pathogen-associated molecular pattern (PAMP) that triggers immune responses through cytosolic sensors. Immune cells encounter both PGN polymers and hydrolyzed muropeptides during infections, and primary human innate immune cells respond better to polymeric PGN than the minimal bioactive subunit muramyl dipeptide (MDP). While MDP is internalized through macropinocytosis and/or clathrin-mediated endocytosis, the internalization of particulate polymeric PGN is unresolved. We show here that PGN macromolecules isolated from *Bacillus anthracis* display a broad range of sizes, making them amenable for multiple internalization pathways. Pharmacologic inhibition indicates that PGN primarily, but not exclusively, is internalized by actin-dependent endocytosis. An alternate clathrin-independent but dynamin dependent pathway supports 20–30% of PGN uptake. In primary monocytes, this alternate pathway does not require activities of RhoA, Cdc42 or Arf6 small GTPases. Selective inhibition of PGN uptake shows that phagolysosomal trafficking, processing and downstream immune responses are drastically affected by actin depolymerization, while dynamin inhibition has a smaller effect. Overall, we show that polymeric PGN internalization occurs through two endocytic pathways with distinct potentials to trigger immune responses.

## 1. Introduction

Recognition and cellular processing of invading pathogens and/or pathogen-associated molecular patterns (PAMPs) are critical for mounting appropriate immune responses during infections. Many pathogens have developed mechanisms to hijack endocytic machineries to either avoid recognition and internalization by immune cells, or to redirect their intracellular trafficking away from the lysosomal compartment [1]. In response, immune cells maintain and use a diverse repertoire of partially overlapping internalization pathways, including phagocytosis, macropinocytosis, dynamin-dependent and dynamin-independent pathways [2,3,4], that allows them to sample the environment even when one or more of the molecular components are targeted by virulence factors. Understanding molecular disturbances of endocytic pathways during infections could elucidate immune impairment and provide new targets for infection prophylaxis [5].

Peptidoglycan (PGN) is a ubiquitous component of the bacterial cell wall, abundantly expressed by Gram-positive (Gram+) bacteria. It is a polymeric macromolecule comprised of linear glycan repeats of N-acetylglucosamine-β(1,4)-N-acetylmuramic acid disaccharides, crosslinked by intercalating small peptides. PGN diversity resides in variations in the stem peptides and post synthetic modifications of glycan strands [6] which, in turn, can modulate downstream immune recognition and responses [7,8,9]. PGN and/or its bioactive fragments are recognized by a variety of immune sensors, including serum opsonins [10,11,12], peptidoglycan recognition proteins (PGLYRPs) [13] and intracellular sensors such as nucleotide-binding oligomerization domain (NOD) proteins NOD1 and NOD2 [9,14] and inflammasomes NLRP1 [15] and NLRP3 [7]. In general, PGN supports proinflammatory immune responses during acute Gram-positive (Gram+) [12,16] and Gram-negative (Gram−) infections [17] and chronic inflammatory pathologies after disruption of intestinal permeability [18,19].

Given the cytosolic distribution of NODs and inflammasomes, PGN, either polymeric macromolecules and/or monomer subunits, have to be internalized by immune cells [20]. The internalization pathway likely depends on how PGN is presented to immune cells. During growth, both Gram+ and Gram− bacteria release PGN subunits into the environment during continuous remodeling of the cell wall. These fragments, likely uncoupled peptidyl-disaccharides biosynthetic modules [21,22] or hydrolyzed muropeptides [23], are internalized by clathrin and dynamin-mediated endocytosis (CME) [24] and transported to cytosol by endolysosomal peptide transporters [25]. Many bacteria, both Gram− and Gram+, also release membrane vesicles (BMVs) containing cell wall components [26]. While their immune recognition and endocytosis is not fully understood, BMVs trigger NOD-dependent immune responses indicative of PGN processing [27,28]. Furthermore, immune-mediated extracellular killing of pathogens [29,30] will lead to fragmentation and release of bacterial cell wall components including polymeric PGN and hydrolyzed muropeptides. We previously observed that human immune cells respond better to polymeric peptidoglycan compared to muramyl-dipeptide (MDP) subunits [31], indicating efficient uptake of these macromolecules. In this study, we explored the internalization mechanisms of polymeric PGN in primary human monocytes and show that, in contrast to MDP, PGN macromolecules are mainly internalized through actin-dependent phagocytosis, with a clathrin-independent but dynamin-dependent alternate pathway responsible for 20–30% of the uptake. Selective inhibition of these endocytic pathways impairs phagolysosome trafficking and reduces immune responses to PGN.

## 2. Materials and Methods

### 2.1. Materials

Cell culture RPMI-1640 media and supplements were from either ATCC (Manassas, VA, USA) or Corning (Manassas, VA, USA). Cell culture grade Hank’s buffer salt solution (HBSS), Dulbecco’s phosphate buffer saline (DPBS), dimethyl sulfoxide (DMSO), FITC (fluorescein isothiocyanate) and Histopaque-1077 were from Millipore-Sigma (St. Louis, MO, USA). Bovine serum albumin (BSA), MACS grade, was from Miltenyi Biotec (Auburn, CA, USA). Peptidoglycan (PGN) was isolated from *Bacillus anthracis* strain Sterne BA781 (Δlef243/Δcya244/ΔpagA242) obtained through the NIH Biodefense and Emerging Infections Research Resources Repository (NR-9401), National Institute of Allergy and Infectious Diseases, National Institutes of Health. The Trypticase Soy Broth was from BD Biosciences (San Jose, CA, USA). Bacteria and PGN were labeled with either FITC or biotin (EZ-link Sulfo-NHS-LC-Biotin, Pierce Biotechnology, Rockford, IL, USA). Sizing fluorescent calibrator nanobeads were from Spherotech (Lake Forest, IL, USA). The endocytic inhibitors latrunculin B (Lat.B), hydroxy-dynasore (OH-Dyn.), Y16, rhosin, ML141 and secin H3 were from EMD Millipore (Billerica, MA, USA), ES9-17 was from Sigma, and NAV 2729 was purchased from Tocris (Minneapolis, MN, USA). For flow cytometry, we used phycoerythrin (PE) or PE-Cyanine7 labeled anti-human CD14 (clone 61D3), FITC labeled anti-human TNF (clone MAb11) and allophycocyanin (APC) labeled anti-human TF/CD142 (clone HTF-1). All cytometry monoclonals and isotype controls were from eBioscience (San Diego, CA, USA). Viability stains, either Zombie Violet or Zombie Aqua, were from BioLegend (San Diego, CA, USA). Trypan Blue (4%) was from Gibco (Waltham, MA, USA). For immunofluorescence microscopy, we used biotinylated mouse anti-human CD14 monoclonals (clones 61D3, Novus Biologicals, Centennial, CO, USA; Tuk4, Invitrogen, Carlsbad, CA, USA; and MEM-15, GeneTex, Irvine, CA, USA), rabbit monoclonal anti-human lysosomal-associated membrane protein 1 (LAMP1/CD107a, clone 107, Novus Biologicals, Centennial, CO, USA), cyanine3-labeled streptavidin and cyanine5-labeled donkey anti-rabbit IgG (both from Jackson ImmunoResearch, West Grove, PA, USA). Saponin and the nuclear counterstain DAPI (4′,6-diamidino-2-phenylindole) dilactate were from Millipore-Sigma (St. Louis, MO, USA), while the ProLong Gold mounting medium was from Life Technologies (Carlsbad, CA, USA). All other chemicals were ACS or cell culture grade and purchased from either Sigma or Fisher Scientific (Waltham, MA, USA).

### 2.2. Analysis of Primary Human Peripheral Blood Mononuclear Cells

Studies on peripheral blood mononuclear cells (PBMCs) were conducted based on a protocol approved by the Institutional Review Board at the Oklahoma Medical Research Foundation (protocol number 19-11) and in accordance with the Declaration of Helsinki. Voluntary participants self-enrolled, were informed of study aims and procedures, and gave written informed consent. Samples were de-identified and coded by the study phlebotomist before experiments. PBMCs were isolated by density gradient centrifugation using Histopaque-1077, washed twice with HBSS and transferred to RPMI-1640 media supplemented with glucose and glutamine. PBMCs were pre-treated with endocytic inhibitors for 1 h in the absence of serum in a humidified atmosphere containing 5% CO_2_, and the inhibitors were maintained throughout the experiment. For internalization studies, treated cells were incubated with pre-opsonized particles, either PGN or heat-killed *Bacillus anthracis* (hk*Ba*), in a thermostated benchtop water bath at 37 °C for 30 min. For activity assays, cells were stimulated with PGN for 6 h at 37 °C in a humidified atmosphere containing 5% CO_2_.

### 2.3. Bacteria Strain, Peptidoglycan Purification and Labeling

*Bacillus anthracis* strain Sterne BA781 was grown in trypticase soy broth, heat inactivated for 1 h at 70 °C and used at 1 × 10^7^ cfu equivalents/mL [32,33]. Anthrax PGN was purified from the BA781 Sterne strain parental bacteria according to protocols detailed previously [8,20,34]. Purified PGN preparations were free of TLR2 and TLR4 agonists [8] and supported internalization-dependent proinflammatory and procoagulant responses in primary human monocytes [11,32].

PGN and hk*Ba* particles were amine labeled using either FITC (Millipore-Sigma, St. Louis, MO, USA) or EZ-link Sulfo-NHS-LC-Biotin (Pierce Biotechnology, Waltham, MA, USA) for 1 h at room temperature in carbonate buffer of pH 9.0. Particles were collected by centrifugation, quenched with 0.1 M glycine, then washed extensively with HBSS and stored in HBSS.

### 2.4. Sizing of PGN Polymers Purified from Bacterial Cell Wall

We quantified the size of polymeric PGN macromolecules by either flow cytometry or scanning electron microscopy (SEM). For nanoscale cytometry, we optimized light scatter voltages and detector thresholds for visualization of submicron events using a mixture of calibrated fluorescent nanobeads with median sizes ranging from 0.13 µm to 1.35 µm (Spherotech, Lake Forest, IL, USA). As reported by other investigators [35], side scatter (SSC) detection provided a better discrimination of submicron particles as compared to forward scatter (FSC), and we subsequently used median SSC quantification for sizing. We generated a “sizing” standard curve by plotting median SSC versus the size of calibrator nanobeads. The best fit polynomial curve (third order polynomial function, R^2^ = 0.9998–1.0) was subsequently used to interpolate the size of PGN particles. Data were acquired on LSRII (BD Biosciences, San Jose, CA, USA) or Cytek Aurora (Cytek Biosciences, Fremont, CA, USA) cytometers.

The size of PGN particles was confirmed by SEM. Biotinylated PGN or hk*Ba* particles were deposited on streptavidin-coated Thermanox coverslips (Electron Microscopy Sciences, Hatfield, PA, USA) for 1 h at room temperature. Samples were fixed with a mixture of 2% paraformaldehyde and 2.5% glutaraldehyde in 0.1 M sodium cacodylate buffer (pH 7.2), followed by 1.0% osmium tetroxide in cacodylate. After stepwise dehydration through a discontinuous 30–100% ethanol gradient, specimens were chemically dried in hexamethyldisilazane for 5 min, air dried in a desiccator for 30 min and sputter coated with gold using a Jeol DII-29010SCTR smart coater (Jeol USA Inc, Peabody, MA, USA). Coated specimens were viewed using a Jeol JCM-6000Plus scanning electron microscope under high vacuum and an accelerating voltage of 15 kV. Orthogonal axes (length and width) were measured within the acquisition software (JCM-6000Plus, Version 1.6.0) for at least 50 independent particles.

### 2.5. Monocyte Internalization of FITC-Labeled PGN and/or hkBa

For internalization studies, freshly isolated PBMCs were left untreated, mock-treated with DMSO or treated with endocytic inhibitors for 1 h at 37 °C in a 5% CO_2_ humidified atmosphere in the absence of serum. PBMCs were then chilled for 10 min on ice before the addition of FITC labeled particles, either PGN or hk*Ba* pre-opsonized with pooled normal human serum. For cytometry analysis, a fluorescence minus one (FMO) reaction using unlabeled particles, either PGN or hk*Ba*, was run in parallel for each experimental condition. Internalization was allowed to proceed for 30 min at 37 °C in a benchtop thermostated water bath, then stopped at endpoint by transferring cells to ice. PBMCs were subsequently stained on ice with PE-labeled anti-human CD14 and the Zombie Violet viability stain, the excess viability dye was quenched with 1% BSA and the cells were washed and resuspended in a known volume of HBSS. Two cytometry acquisitions were performed for each sample: an initial acquisition for 1 min before Trypan Blue (TB) quenching, followed by addition of one volume of 2% TB solution to quench surface fluorescence (30 s on ice) and acquisition of TB quenched samples for 2 min. For each run, TB’s quenching coefficient was determined using surface FITC-labeled calibrator beads (Spherotech, Lake Forest, IL, USA) under the same acquisition protocol. Throughout the study, TB quenched the calibrator beads by roughly 90% (median 92.41%, range 87.42–95.15%). Data were acquired on a LSRII cytometer using BD FACSDiva software (Version 9.0; BD Biosciences, San Jose, CA, USA) and subsequently analyzed using FlowJo (version 10.8.1; FlowJo LLC, Ashland, OR, USA) and Prism (Version 9.3.1; GraphPad Software, San Diego, CA, USA).

During post-acquisition data processing, the frequency of phagocytic monocytes (FITC+CD14+), defined as CD14+ PBMCs that internalized FITC labeled particles, was assessed in FlowJo by gating above 99.5% events of the paired FMO controls. To quantify the fluorescence intensity of internalized particles, we subtracted the expected residual surface signals from the experimentally observed fluorescence intensity after TB quenching. Assuming that TB quenches only the surface fluorescence, as observed using calibrator beads, with minimal to no internalization of the dye during the quenching step (30 s on ice before acquisition), then:

MFI_Internal_ = (MFI_TB+_ − K × MFI_TB−_)/(1 − K) (1)

where MFI_Internal_ represents the adjusted internalized fluorescence, MFI_TB−_ and MFI_TB+_ represent measured fluorescence intensities before and, respectively, after TB quenching, and K represents the residual quenched surface fluorescence assessed using calibrator beads (K = bead_TB+_/bead_TB−_).

To reduce the variability between donors, paired normalization of particle uptake was performed by considering uptake by DMSO-treated PBMCs (mock challenge) as 100% response, while unstimulated controls were set at 0. When titrating the effect of latrunculin B on PGN uptake, a four-parameter logistic curve was fitted to normalized responses at increasing doses of latrunculin to estimate the relative IC_50_ and maximum effect of the inhibitor.

### 2.6. Fluorescence Microscopy Analysis of FITC-PGN Uptake by Monocytes

Freshly isolated PBMCs were seeded on poly-L-lysine coated glass coverslips in a 24-well plate, and monocytes were allowed to adhere for 1 h in the absence of serum. PBMCs were pre-treated with either DMSO (mock challenge), latrunculin B or hydroxy-dynasore, both at 20 µM, for 1 h before addition of pre-opsonized PGN-FITC. Internalization of PGN-FITC progressed for 30 min at 37 °C, and was stopped by paraformaldehyde fixation (4% final concentration). Cells were washed, surface stained with a mixture of three biotinylated monoclonals anti-human CD14 (clones 61D3, Tuk4 and MEM-15, each at 2 µg/mL) for 1 h at room temperature, permeabilized with saponin and stained for the lysosomal associated protein 1 (LAMP1) using a rabbit monoclonal antibody (clone 107, Novus Biologicals, 5 µg/mL). Cells were washed and incubated with detection reagents, Cy3-labeled streptavidin and Cy5-labeled donkey anti-rabbit IgG (Jackson ImmunoResearch, West Grove, PA, USA), fixed post-stain for 15 min with 4% paraformaldehyde, counterstained with 5 µg/mL DAPI dilactate (Sigma, St. Louis, MO, USA) for 5 min at room temperature, washed and mounted in ProLong Gold (Life Technologies, Carlsbad, CA, USA). Mounted specimens were cured for at least 24 h at room temperature, protected from light, sealed and stored at 4 °C until imaging.

Confocal images were acquired on a Nikon Ti2 inverted microscope (Nikon Instruments Inc., Melville, NY, USA) equipped with a four-laser excitation unit and a C2 Plus confocal scanning head using a Nikon Apo 60× NA 1.40 oil immersion objective. Z-stack images were collected at 0.5-μm steps with confocal parameters selected to minimize the thickness of the calculated optical section using the NIS-Elements acquisition software (version 4.60, Nikon Instruments Inc., Melville, NY, USA). Post-acquisition, confocal datasets were processed in Imaris software (Version 9.5.1, Oxford Instruments, Concord, MA, USA) and presented as flattened maximum intensity projections of the z-stacks. The monocyte envelope was estimated by 3D rendering of CD14 staining in Imaris. For PGN/LAMP1 colocalization, z-stacks were segmented based on CD14 staining to limit analysis to cell-associated PGN. Individual PGN and LAMP1 signals were thresholded to eliminate background fluorescence and co-distribution of their respective staining was assessed in Imaris. A minimum of five independent z-stack datasets with at least 30 cumulative phagocytic monocytes were analyzed for each donor per experimental condition, and the mean LAMP1 colocalized volume as a fraction of total cellular PGN volume was used for comparisons between experimental groups.

### 2.7. Flow Cytometry Analysis of Procoagulant and Proinflammatory Responses to PGN

Freshly isolated PBMCs were left untreated, mock-treated with DMSO or treated with endocytic inhibitors in the absence of serum as above, then stimulated with 20 µg/mL pre-opsonized PGN in the presence of brefeldin A (3 µg/mL) for 6 h at 37 °C in a humidified atmosphere containing 5% CO_2_. After stimulation, cells were transferred to ice, stained for surface markers and viability (Zombie Aqua fixable viability stain, BioLegend, San Diego, CA, USA), fixed with 4% paraformaldehyde and stained for intracellular inducible antigens after saponin permeabilization. Dead cells were excluded based on viability stain positivity and monocytes were identified based on size, scatter and CD14 expression. Monocyte procoagulant responses were defined by tissue factor (TF/CD142) induction, while proinflammatory responses were defined by TNF. Data were collected on a LSRII cytometer using BD FACSDiva software (Version 9.0).

### 2.8. Data Analysis and Representation

Analysis of flow cytometry data and histogram overlays was performed in FlowJo (Version 10.8.1). Thresholds were set using either FMO (fluorescence minus one) controls for bacteria and PGN internalization, or isotype immunostaining run in parallel. Microscopy analysis was performed in Imaris software (Version 9.5.1). Polynomial curve fitting, interpolation of sizes from standard curves, normalizations and statistical analysis were all performed in Prism (Version 9.3.1). Differences between experimental groups were analyzed by repeated measures (RM) analysis of variance (ANOVA) with Geisser-Greenhouse correction for sphericity and Holm-Sidak’s multiple comparisons test, unless otherwise noted. During latrunculin titration, the range of the inhibitor was extended from 20 to 40 µM in 10 out of 14 donors analyzed. As a result, some pairwise comparisons had missing values and the dataset was analyzed by a mixed-effects, restricted maximum likelihood model with Geisser–Greenhouse and Holm-Sidak’s corrections. For normalized data, we assessed significant deviations from the reference value of 100 set by the normalization process using one sample *t* test. The threshold for statistical significance was set at *p* < 0.05 and, unless detailed otherwise, adjusted *p* values are conventionally represented across graphs (* *p* < 0.05, ** *p* < 0.01, *** *p* < 0.001, **** *p* < 0.0001).

Individual donor responses along with median (dashed line), IQR (interquartile range, floating bars) and range (whiskers) are graphically depicted for either the frequency of responsive CD14+ monocytes (either phagocytic, TF+ or TNF+ monocytes) or the relative fluorescence intensity in CD14+ monocytes (individual values represent geometric mean of relative fluorescence intensities, gMFI). For comparisons of uptake between different particles (PGN vs. hk*Ba*) or screening of multiple endocytic inhibitors, a summary representation depicting the mean ± standard deviation was employed for clarity. Individual panels were generated with Prism (Version 9.3.1) except for histogram overlays (FlowJo, Version 10.8.1) and confocal micrographs (Imaris, Version 9.5.1), and figures were collated in Adobe Illustrator (Version 26.0.2, Adobe Inc., San Jose, CA, USA).

## 3. Results

### 3.1. Sizing of PGN Macromolecules Purified from the Cell Wall of Bacillus Anthracis

**Figure 1:**
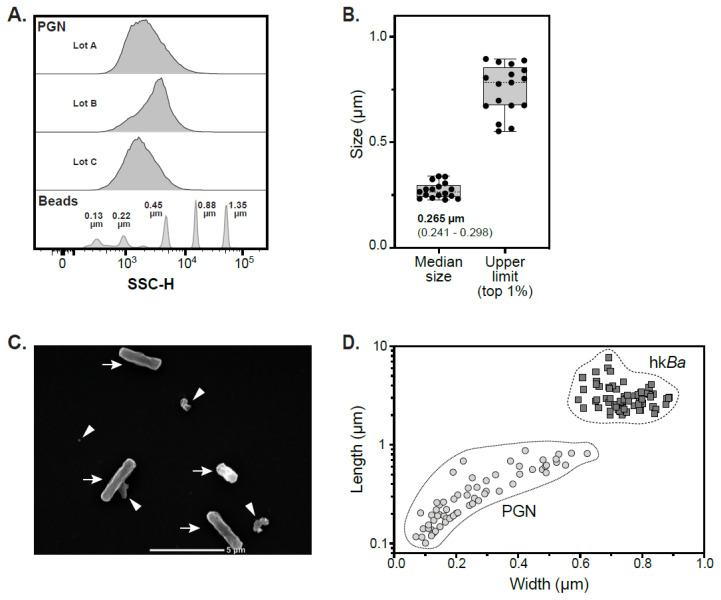
Size quantification of PGN macromolecules purified from the bacterial cell wall by nanoscale flow cytometry and scanning electron microscopy (SEM). (**A**) Histogram overlay of SSC measurements from 3 independent PGN lots depicting broad particle size distribution. For comparison, median SSC for nanobeads of known sizes used to calibrate the cytometry acquisition and generate the sizing standard curve is shown at the bottom. (**B**) Floating bar representation of sizes of PGN macromolecules from 17 independent purification lots depicting the median size (left) and the upper range (top 1%, right) of PGN particles. The estimated median and interquartile range (IQR) of PGN particles is noted in the inset. The lower limit of PGN particles (not shown) was below the lower limit of quantitation (see details in text). (**C**) Scanning electron micrograph of PGN particles (arrowheads) and heat-killed *Bacillus anthracis* (arrows). (**D**) Distribution of PGN and *Bacillus anthracis* (hk*Ba*) particles depicted based on orthogonal measurement of individual particles. For clarity, length is shown on a logarithmic scale while width is shown on a linear scale. Single lot, individual PGN particles (*n* = 64, circles) and hk*Ba* (*n* = 73, squares) are shown.

We previously reported that primary human innate immune cells efficiently recognize particulate bacterial PGN [10,11,33], and they internalize and process particulate PGN better than monomeric muramyl dipeptide (MDP) fragments [20,31]. Since the dimensions of the internalization cargo may limit the repertoire of endocytic pathways available for use, we estimated the size of polymeric peptidoglycan macromolecules purified from the bacterial cell wall of *Bacillus anthracis* by either calibrated nanoscale cytometry or scanning electron microscopy (SEM). For flow cytometry, we used calibrated nanobeads of known median size to optimize acquisition of submicron particles and generate a polynomial fit standard curve (third order polynomial function) from which PGN sizes were interpolated. As shown in Figure 1A,B, purified peptidoglycan polymers display broad sizes. While the median size among 17 independent anthrax PGN lots was similar (0.265 µm median, IQR 0.241–0.298 µm, range 0.228–0.339 µm), we observed higher variability for the largest 1% of PGN particles in these preps (median 0.784 µm, IQR 0.674–0.856 µm, range 0.552–0.896 µm). In a subset of four PGN preparations that were FITC-labeled to differentiate smaller particles from instrument noise during acquisition, the lower 1% of PGN particles was below the lowest limit of quantitation defined by the smallest calibrator bead (0.13 µm).

To test the accuracy of nanoscale flow cytometry estimates we measured the orthogonal axes of individual PGN particles after SEM imaging. SEM measurements of parental bacteria are shown for comparison (Figure 1C,D). As seen with flow cytometry, there was a wide distribution of sizes for PGN particles measured by SEM. While the overall length of PGN particles varied between 0.101 and 0.874 µm, the 0.268 µm median length was in close agreement with the above estimates. The width of PGN particles varied between 0.070 and 0.623 µm, with a median of 0.205 µm. For comparison, the rod-shaped parental *Bacillus anthracis* strain Sterne BA781 displayed a 2.87 µm median length (IQR 2.4–3.36 µm, range 2.0–7.69 µm) and 0.739 µm width (IQR 0.697–0.799, range 0.594–0.887 µm).

Overall, our data show a broad size range for anthrax PGN polymers within the bacterial cell wall, with a median of 0.265 µm. There is a good consistency between nanoscale flow cytometry estimates and the particle length observed by SEM, reflecting the accuracy of the method. These results indicate that PGN macromolecules released from the bacterial cell wall are similar or bigger than exosomes and extracellular vesicles released by mammalian cells, and their size could limit the endocytic pathways available for internalization by immune cells.

### 3.2. Pairwise Comparison of PGN and hkBa Internalization by Human Monocytes

**Figure 2:**
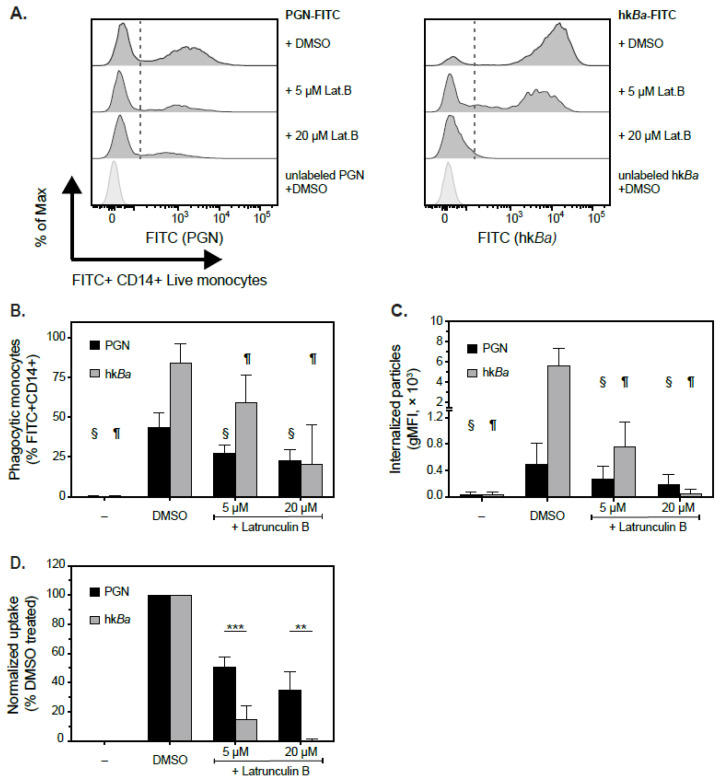
Comparison of latrunculin inhibition of PGN and hk*Ba* internalization by primary human monocytes. (**A**) Representative histogram overlays of a median responsive individual depicting paired internalization of PGN-FITC (left) and hk*Ba*-FITC (right) in CD14+ monocytes pretreated with DMSO (top) or either 5 µM or 20 µM latrunculin B (Lat.B). For comparison, the internalization of unlabeled PGN in DMSO-treated monocytes is shown in the bottom panel, while the threshold used to assess phagocytic cells is depicted graphically as a segmented line. (**B**,**C**) Summary representation of changes in monocyte internalization of FITC labeled particles, either PGN (black bars) or hk*Ba* (grey bars), after latrunculin treatment. Data depict mean ± standard deviation of phagocytic monocytes (%FITC+ CD14+; (**B**)) or adjusted internalized fluorescence intensity (geometric mean fluorescence intensity, gMFI; (**C**)) of 6 independent experiments. Differences between groups were analyzed by repeated-measures two-way ANOVA with Holm-Sidak’s multiple comparison correction (§ depicts significant changes, *p* ≤ 0.05, compared to PGN-FITC internalization by DMSO-treated monocytes; ¶ depicts significant changes, *p* ≤ 0.01, compared to hk*Ba*-FITC internalization by DMSO-treated monocytes). (**D**) Latrunculin effects on PGN and hk*Ba* internalization after pairwise normalization to particle uptake in DMSO treated cells by the same donor. Statistically significant differences between latrunculin’s effects on PGN vs. hk*Ba* uptake are depicted conventionally (** *p* < 0.01, *** *p* < 0.001).

Given the dimensions of PGN macromolecules, we hypothesized that innate immune cells internalize PGN through phagocytosis, an endocytic process that critically requires reorganization of actin cytoskeleton. We therefore tested whether actin depolymerizing agents [36] block the internalization of PGN and/or hk*Ba* by monocytes. Whilst we reported that immune responses to PGN are sensitive to cytochalasin D [8,32], preliminary internalization experiments indicated that latrunculins are more potent than cytochalasin D, with latrunculin B slightly more potent than latrunculin A (data not shown). Latrunculin B was subsequently used as the actin depolymerizing agent for the rest of the study. As shown in Figure 2, latrunculin B reduced the internalization of PGN in a concentration dependent manner, albeit less than the inhibition of hk*Ba* uptake. Incubation of PBMCs with PGN-FITC for 30 min at 37 °C led to particle uptake by 43.9 ± 9.1% CD14+ monocytes (range 35–58.3% phagocytic monocytes; Figure 2B), which was significantly reduced to 27.5 ± 5.3% in the presence of 5 µM latrunculin B (*p* = 0.001, RM two-way ANOVA) and to 22.7 ± 6.9% by 20 µM latrunculin (*p* = 0.001). Similarly, the fluorescence intensity of internalized particles was reduced from 502.6 RFU (relative fluorescence units) in DMSO treated cells to 278.8 RFU in PBMCs treated with 5 µM (*p* =0.033) and 191.7 RFU in the presence of 20 µM latrunculin (*p* = 0.033; Figure 2C). Under similar conditions, monocytes internalized hk*Ba* better, with 84.3 ± 12.2% phagocytic monocytes observed on average (range 65.4–96.2% phagocytic monocytes) after 30 min in the absence of the inhibitor. Bacteria internalization was significantly reduced by latrunculin B (Figure 2B), both at 5 µM (59.4 ± 17.3%, *p* = 0.0004 compared to DMSO treatment) and 20 µM inhibitor (20.5 ± 24.7%, *p* = 0.0004). The intensity of internalized hk*Ba* dropped from 5645 ± 1735 RFU in DMSO treated cells to 755 ± 383 RFU in 5 µM latrunculin-treated (*p* = 0.002) and 62.5 ± 52.6 RFU (*p* = 0.0015 compared to DMSO treated cells) in the presence of 20 µM inhibitor, with the latter not significantly different from background monocyte autofluorescence (39.5 ± 41.3 RFU). After normalization (Figure 2D), pairwise analysis of latrunculin inhibition suggests that hk*Ba* uptake is more sensitive to latrunculin B than PGN uptake at both 5 µM (85.1 ± 9.0% reduction in hk*Ba* uptake compared to 49 ± 6.6% reduction in PGN, *p* = 0.0004, RM ANOVA with Sidak’s multicomparison correction) and 20 µM inhibitor (99.9 ± 1% reduction in hk*Ba* uptake compared to 65.3 ± 12.5 reduction in PGN, *p* = 0.0045). These data suggest that, while hk*Ba* uptake is exclusively actin-dependent and effectively blocked by 20 µM latrunculin B, monocytes internalize PGN preferentially, but not exclusively, through actin-dependent mechanisms.

### 3.3. Actin-Dependent Mechanisms of PGN Uptake

**Figure 3:**
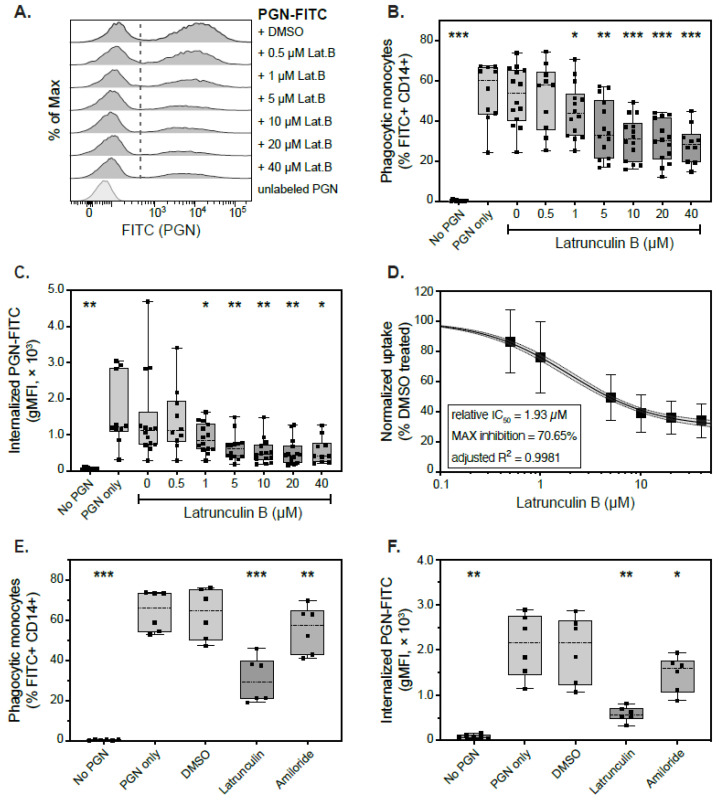
Monocyte uptake of PGN preferentially, but not exclusively, employs actin-dependent pathways. (**A**) Representative histogram overlay of a median responsive individual depicting titration of the latrunculin B inhibition of PGN uptake. For comparison, the internalization of unlabeled PGN in DMSO-treated monocytes is shown in the bottom panel, while the threshold used to assess phagocytic cells is depicted graphically as a segmented line. (**B**,**C**) Graphical representation of PGN uptake sensitivity to latrunculin B in 14 independent individuals, depicted as either phagocytic monocytes (**B**) or geometric mean of fluorescence intensity of internalized PGN-FITC particles (**C**). (**D**) Mathematical modelling of latrunculin B inhibition of PGN-FITC uptake. After pairwise normalization of PGN uptake, a four-parameter logistic curve (continuous line) was fitted by least square regression, and the relative IC_50_ and predicted maximum inhibition with latrunculin B were extracted from the model and shown in the inset. Dotted lines and shaded area depict the 95% confidence interval of the polynomial fit. (**E**,**F**) Pairwise comparison of PGN uptake sensitivity to latrunculin B (20 µM) and the macropinocytosis inhibitor amiloride (1 mM) in 6 independent donors, depicted as either phagocytic monocytes (**E**) or geometric mean fluorescence intensity of internalized PGN-FITC particles (**F**). Statistically significant differences compared to PGN uptake in the absence of the inhibitor in panels (**B**,**C**,**E**,**F**) are depicted conventionally (* *p* < 0.05, ** *p* < 0.01, *** *p* < 0.001).

Since latrunculin B did not fully inhibit PGN uptake, we hypothesized that either (1) actin depolymerization may be incomplete but still sufficient to internalize smaller submicron particles as opposed to larger bacteria, or (2) PGN uptake may occur through actin-independent mechanisms. To test these possibilities, we modeled inhibition of PGN uptake after latrunculin titration over an extended concentration range (0–40 µM) in a subset of 14 donors. As shown in Figure 3, latrunculin B did not fully block PGN internalization at any of the concentrations tested. Both the frequency of phagocytic monocytes (Figure 3B) and the intensity of internalized fluorescent particles (Figure 3C) were significantly reduced starting at 1 µM inhibitor. At the highest concentration of latrunculin B tested, phagocytic monocytes were reduced by roughly 50% (28.3% compared to 54.1% PGN+CD14+ monocytes in the absence of inhibitor; *p* = 0.0009, mixed-effects model with Holm-Sidak’s multicomparison correction) and the internalized fluorescence was reduced by 66% (557 ± 361 RFU compared to 1595 ± 965 RFU in the absence of inhibitor; *p* = 0.036). After pairwise normalization to PGN uptake in control (DMSO) treated cells, latrunculin responses were fitted to a four-parameter logistic curve by least square regression (adjusted R^2^ = 0.9981). Mathematical modeling estimated a relative latrunculin IC_50_ of 1.93 µM and plateauing of inhibition at 29.35%, resulting in a maximum latrunculin-mediated inhibition of roughly 70% (Figure 3D). These data indicate that latrunculin B does not fully block the uptake of PGN particles, and actin-independent mechanisms could provide alternate internalization pathways.

Actin-depolymerizing agents, such as latrunculin, affect two main internalization pathways: phagocytosis and macropinocytosis. To assess the relative contribution of macropinocytosis to PGN internalization, we measured PGN uptake in the presence of amiloride, a macropinocytosis inhibitor. As shown in Figure 3E,F, PGN uptake was less sensitive to amiloride (1 mM) than latrunculin B (20 µM). Amiloride induced a small reduction in both the frequency of phagocytic monocytes (55.3% compared to 63.1% in the absence of inhibitor; *p* = 0.0032, RM ANOVA with Holm-Sidak’s correction) and the fluorescence intensity of the internalized particles (1467 ± 395 RFU compared to 2019 ± 738 RFU in the absence of inhibitor; *p* = 0.0413). Pairwise, latrunculin B was a more potent inhibitor than amiloride (30.4% phagocytic monocytes, *p* = 0.0001 compared to amiloride treatment and, respectively, 583 ± 168 RFU internalized MFI, *p* = 0.003 compared to amiloride). These data indicate that PGN particles are primarily internalized through actin-dependent phagocytosis and not macropinocytosis.

### 3.4. Actin-Independent Internalization of PGN Uptake

**Figure 4:**
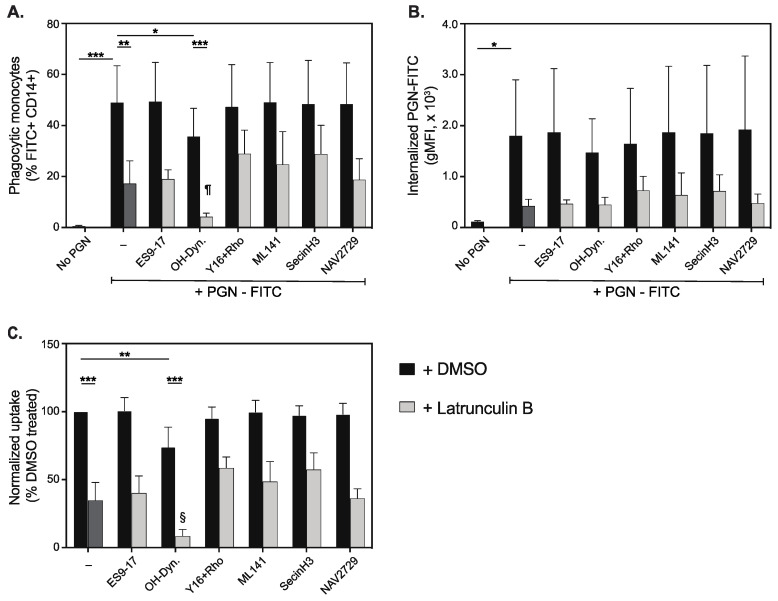
Actin-independent internalization of PGN particles by primary human monocytes. Summary representation (mean ± SD, *n* = 8) of monocyte internalization of FITC labeled PGN in the presence of endocytic inhibitors, by themselves (black bars) or in combination with 20 µM latrunculin B (light grey bars). The single inhibition with latrunculin is depicted as a dark grey bar in the absence of additional inhibitors. Panels depict frequency of phagocytic monocytes after 30 min incubation with PGN-FITC particles at 37 °C (**A**), the adjusted fluorescence intensity of internalized particles (gMFI, (**B**)) and the relative effect of inhibitors after pairwise normalization to uptake in mock (DMSO) treated cells (**C**). Statistically significant differences between relevant groups are depicted conventionally (* *p* < 0.05, ** *p* < 0.01, *** *p* < 0.001), except for panel (**A**,**C**), where ¶ (*p* = 0.0363) and § (*p* = 0.0074) depict significant changes when the combined latrunculin B and hydroxy-dynasore treatment is compared to latrunculin alone.

To assess the contribution of actin-independent mechanisms to PGN uptake by primary human monocytes, we screened a panel of pharmacologic inhibitors of endocytic pathways, either individually or in combination with latrunculin inhibition (Figure 4). For this purpose, we tested inhibitors of clathrin (ES9-17 [37], 20 µM), dynamin (hydroxy-dynasore [38], 20 µM), RhoA (synergistic inhibitors Y16 and rhosin [39], 10 µM each), Cdc42 (ML141 [40], 10 µM) and Arf6 (Secin H3 [41], 50 µM, and/or NAV 2729 [42], 10 µM), all of which contribute to overlapping repertoires of internalization pathways. When using single inhibitors, DMSO was added to maintain vehicle concentration constant.

As seen in Figure 4, hydroxy-dynasore (OH-Dyn.) was the only pharmacological agent that reduced PGN-FITC uptake in this panel, either by itself or in combination with latrunculin B. Hydroxy-dynasore reduced the frequency of phagocytic monocytes after 30 min incubation with PGN-FITC at 37 °C, from 49.1 ± 14.4% in control-treated cells to 35.7 ± 11.1% (*p* = 0.0452, RM ANOVA with Holm-Sidak’s multicomparison correction (Figure 4A). The effect was less pronounced than latrunculin, which reduced phagocytic monocytes to 17.2 ± 8.9% (*p* = 0.0021 compared to DMSO treatment) in these donors. The combined treatment with latrunculin and hydroxy-dynasore provided the strongest reduction in phagocytic monocytes, to 4.2 ± 1.4% (*p* = 0.0008 compared to OH-Dyn alone, and *p* = 0.0363 compared to latrunculin B treatment). However, the hydroxy-dynasore mediated reduction in fluorescence intensity of internalized particles (Figure 4B), from 1799 ± 1100 RFU in DMSO-treated cells to 1471 ± 664 RFU, did not pass the statistical threshold. After pairwise normalization to decrease variability between donors (Figure 4C), hydroxy-dynasore inhibition accounted for a 26.1% reduction of PGN-FITC uptake (*p* = 0.0093), while latrunculin B reduced it by 65.4% (*p* < 0.0001). Overall, actin and dynamin dependent internalizations support virtually all PGN uptake, as combined inhibition with latrunculin and hydroxy-dynasore reduces uptake by roughly 90%.

### 3.5. Phagolysosomal Trafficking of Internalized PGN

**Figure 5:**
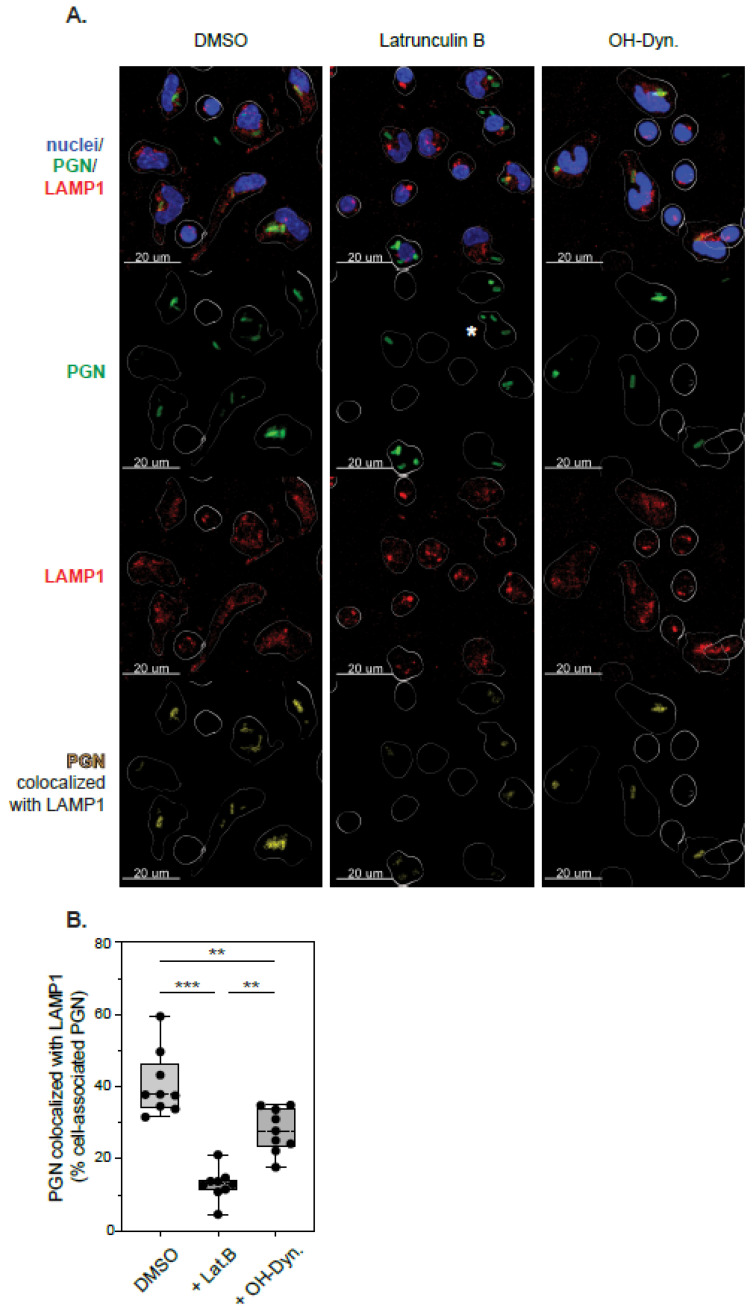
Phagolysosomal trafficking of PGN after inhibition of actin and/or dynamin-dependent internalization. (**A**) Representative confocal micrographs depicting phagolysosomal trafficking of PGN-FITC in a median responsive individual in the absence (left column) or presence of internalization inhibitors (middle and right columns). Top row illustrates maximum intensity projections of confocal z-stacks showing PGN (green), LAMP1 (red) and cell nuclei (blue). Middle rows depict individual PGN (green) and LAMP1 (red) signals in monocytes, while the bottom row shows cell associated PGN that colocalizes with LAMP1 (yellow). In all panels, the monocyte envelope was projected by 3D rendering of CD14 staining using Imaris and is depicted as an intermittent white line to highlight cellular regions. The colocalization signals were generated in Imaris based on the co-distribution of individual PGN and LAMP1 signals in z-stack datasets. Note the reduction in colocalization signals (bottom panels), primarily in latrunculin-treated cells. Internalized PGNs with no associated LAMP1 were consistently observed in latrunculin-treated cells and are highlighted (*) in the middle panel. (**B**) Graphical representation of latrunculin- and dynasore-induced changes in PGN-LAMP1 colocalization in monocytes isolated from 9 independent donors. Individual responses represent the mean volume of cell-associated PGN that colocalizes with LAMP1 from 5 independent confocal z-stacks per donor. Statistically significant differences between experimental groups are depicted conventionally (** *p* < 0.01, *** *p* < 0.001).

Lysosomal processing of polymeric bacterial PGN is needed to release signaling-active subunits responsible for immune activation [20]. Since different internalization pathways may direct cargo to distinct endocytic compartments, we investigated whether actin- and/or dynamin-mediated internalization affect the phagolysosomal targeting of PGN. Freshly isolated PBMCs were seeded on poly-L-lysine coated coverslips to allow monocyte attachment, pre-treated with DMSO (vehicle), latrunculin or hydroxy-dynasore in the absence of serum for 1 h, incubated with PGN-FITC particles for 30 min at 37 °C, then fixed and processed for immunofluorescence microscopy. The phagolysosomal progression of monocyte-associated PGN particles was assessed by colocalization with the lysosomal marker LAMP1/CD107a (lysosomal-associated membrane protein 1). As shown in Figure 5, fluorescent PGN particles associated with monocytes under all experimental conditions tested, although, as previously quantified by flow cytometry, the number of phagocytic cells was reduced in the presence of either inhibitor. CD14 staining coalesced around PGN particles in line with CD14 recognition of PGN carbohydrates [43]. PGN internalization for 30 min at 37 °C led to phagolysosomal targeting of PGN, with roughly 40% of cell associated PGN-FITC particles colocalizing with LAMP1 in the absence of inhibitors (median 37.8%, IQR 34.2–46.4%, range 31.6–59.5%). Inhibition of actin-polymerization by latrunculin reduced PGN phagocytosis and slowed down phagolysosomal trafficking of cell associated PGN, with only 13% PGN-FITC particles colocalizing with LAMP1 after 30 min (median 12.99%, IQR 11.2–14.3%, range 4.6–21.1%; *p* = 0.0002 compared to DMSO-treated cells). Similar to the flow cytometry studies above, dynamin inhibition had a smaller effect on PGN-LAMP1 colocalization (median 27.8%, IQR 23.2–34.9%, range 17.7–34.9%; *p* = 0.0037 compared to DMSO-treated cells and, respectively, *p* = 0.0017 compared to latrunculin treatment). From these studies, we conclude that PGN internalization through actin-dependent phagocytosis supports faster targeting of PGN for lysosomal degradation. Dynamin-internalized PGN endosomes, which become predominant in latrunculin treated cells, either recruit lysosomes more slowly or they are directed towards other endocytic compartments, leading to decreased colocalization with LAMP1 in these timed experiments.

### 3.6. Effect of Endocytic Inhibitors on Immune Responses to PGN

**Figure 6:**
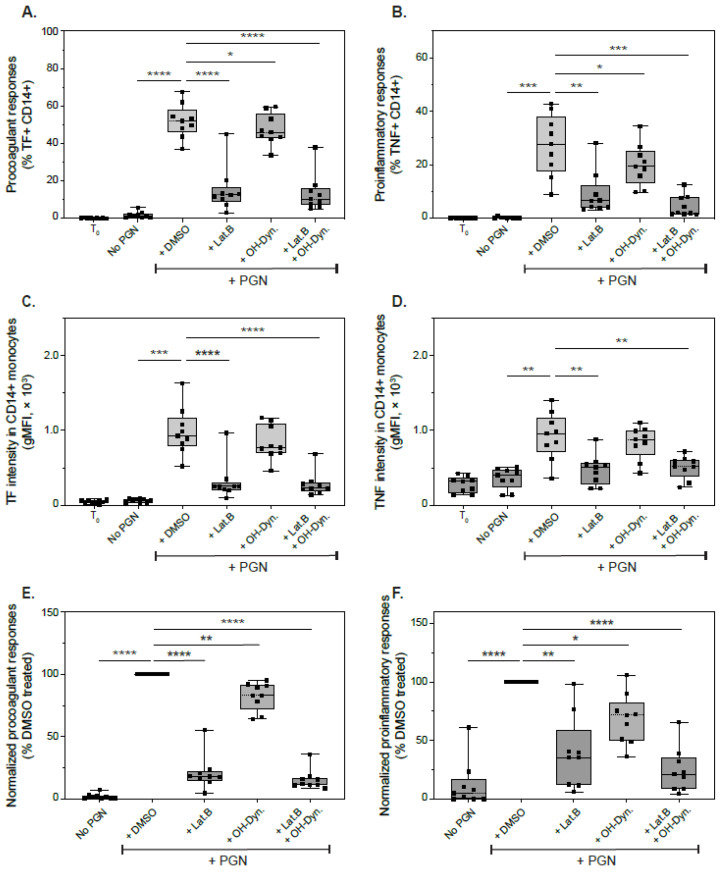
Modulation of immune responses to PGN after inhibition of actin and/or dynamin-dependent internalizations. Primary human PBMCs were mock-treated (DMSO) or treated with latrunculin B (Lat.B) or hydroxy-dynasore (OH-Dyn.) for 1 h, followed by 6 h stimulation with PGN (20 µg/mL) in the presence of brefeldin A. Inducible expressions of TF (**A**,**C**,**E**) or TNF (**B**,**D**,**F**) were assessed by flow cytometry after comparison with a paired unstimulated aliquot fixed at the start of the experiment (T_0_). Data depict frequency of responsive monocytes (**A**,**B**), the geometric mean of fluorescence intensity of inducible antigens (**C**,**D**) and normalized induction of TF and TNF (**E**,**F**) after pairwise normalization to DMSO-treated cells (*n* = 9). Statistically significant differences compared to PGN responses in the absence of the inhibitors (DMSO-treated cells) are depicted conventionally (* *p* < 0.05, ** *p* < 0.01, *** *p* < 0.001, **** *p* < 0.0001; RM ANOVA with Holm-Sidak’s multicomparisons test).

Since latrunculin and hydroxy-dynasore reduced the uptake and intracellular trafficking of PGN particles, we hypothesized they would subsequently affect immune responses to PGN which primarily occur downstream of lysosomal processing. We tested this hypothesis by quantifying monocyte procoagulant and proinflammatory responses after stimulation with 20 µg/mL PGN for 6 h in the presence of brefeldin A to block the cytokine-mediated feedback amplification [32]. In line with this model, latrunculin B and, to a lesser extent, hydroxy-dynasore, reduced both tissue factor (TF) and TNF induction post-challenge (Figure 6).

As illustrated in Figure 6A, the frequency of procoagulant monocytes, defined as TF+CD14+, was significantly reduced by latrunculin B from 52% (IQR 45.9–58.2%, range 37.1–67.6%), in mock treated cells, to 12.5% (IQR 8.8–16.8%, range 3–45.1%; *p* < 0.0001 RM ANOVA with Holm-Sidak’s post test). Similarly, the intensity of TF signal in monocytes decreased from 928 RFU (IQR 786–1167 RFU, range 523–1632 RFU) to 253 RFU (IQR 205–304 RFU, range 96–965 RFU; *p* < 0.0001). Inhibition of dynamin-dependent internalization had a smaller effect on PGN-induced procoagulant responses and reduced the frequency of TF+CD14+ monocytes to 45.9% (IQR 42.9–56%, range 33.6–59.6%; *p* = 0.0167). The small reduction in monocyte TF intensity (median 769, IQR 695–1093 RFU, range 460–1170) approached, but did not pass, the significance threshold (*p* = 0.0579 compared to DMSO treatment). After pairwise normalization to immune responses in control treated cells (Figure 6E), hydroxy-dynasore reduced TF induction by 17.6% (*p* = 0.0053), while latrunculin reduced TF by 79.1% (*p* < 0.0001). These results closely resemble the effects of the inhibitors on PGN uptake and indicate that procoagulant responses to PGN are internalization-dependent. The combined treatment with both latrunculin B and hydroxy-dynasore inhibited TF induction the most (85% reduction; *p* = 0.0398 when compared to latrunculin alone, and *p* < 0.0001 compared with either OH-Dyn and/or DMSO treatment).

Modulation of monocyte proinflammatory responses, defined by TNF expression, mimicked changes observed for procoagulant responses (Figure 6B,D,F). TNF induction, however, displayed a higher degree of variability between individual donors compared to procoagulant responses. Interestingly, after normalization (Figure 6F), dynasore-inhibition of TNF was more pronounced than TF reduction, resulting in a 31.5% inhibition of TNF (*p* = 0.0112 compared to control treatment), while concomitantly latrunculin reduction of TNF was smaller than that observed for TF: 60.3% compared to 79.1%, respectively. Due to the higher variability in TNF responses between donors, the TNF reduction observed with the combined latrunculin and dynasore treatment (75.4%) was not significantly different than from latrunculin alone (*p* = 0.066). Overall, our results confirm that immune responses to PGN primarily occur following internalization and lysosomal processing, since combined latrunculin and dynasore inhibition reduced proinflammatory and procoagulant responses by 75–85%. Interestingly, the reduction in frequency of activated monocyte after hydroxy-dynasore, in both longer activity assays and shorter timed uptake experiments, seem to indicate that at least a subset of monocytes exclusively utilizes the dynamin pathway to take up this PAMP.

## 4. Discussion

Systemic anthrax is characterized by high bacteremia [44], which leads to an increased burden of both pathogen and pathogen-derived PAMPs. While virulence factors such as the bipartite anthrax toxins contribute to disease progression and pathology, other non-toxigenic PAMPs support clinical anthrax manifestations [45,46,47]. We and others have identified cell wall peptidoglycan (PGN) as a major PAMP supporting septic anthrax pathology [12,16,48]. Anthrax peptidoglycan triggers proinflammatory responses in primary human monocytes, neutrophils [20,32,34,49] and in non-human primates in vivo [12,16], primarily through activation of intracellular NOD receptors [31]. Thus, efficient immune responses to PGN require internalization and lysosomal processing of the macromolecule within phagocytes. Peripheral blood leukocytes, primarily monocytes and neutrophils, respond better to particulate anthrax PGN than MDP [31], which, in part, may be due to better recognition and uptake of the polymeric PGN macromolecule than its hydrolyzed monomers. We are unaware of reports describing the mechanisms of anthrax PGN internalization in primary human innate immune cells until now; however, immune responses to particulate PGN were sensitive to cytochalasin D [8,11,20,32], indicative of actin-mediated internalization. Likewise, latrunculin B in this study inhibited most of the uptake and subsequent immune responses to PGN. Extensive titration and modelling of latrunculin-mediated inhibition revealed that, even at high doses, 10–20× IC_50_, there is a significant, roughly 30%, actin-independent uptake of PGN. We subsequently show this pathway to be dynamin-dependent. The dynamin pathway was not triggered because of the phagocytic blockade since the dynamin inhibitor reduced PGN uptake in the absence of any other inhibitor.

Reorganization of the cortical actin cytoskeleton is a critical early step in both immune-receptor mediated phagocytosis [2] and macropinocytosis [3], the nonspecific uptake of large volumes of extracellular fluid. Macropinocytosis has been shown to support macrophage uptake of MDP [50] as well as the internalization of bacterial cell wall by epithelial cells [51]. In the current study, latrunculin was a more potent inhibitor of PGN uptake than amiloride, a selective inhibitor of macropinocytosis which also inhibits dynamin-dependent fast endophilin-mediated endocytosis (FEME) [52]. In pairwise comparisons, PGN uptake was 3.25-fold more sensitive to latrunculin than to amiloride (range 1.9×–4.6× in individual donors). The small amiloride effect most likely depicts involvement of constitutive macropinocytosis, FEME or a combination of the two. Overall, our data support a model of phagocytic internalization of PGN as the main actin-dependent internalization pathway in primary human monocytes.

Our current observations together with previous reports detailing PGN opsonization requirements [10,11,33], engagement of Fc receptors [10,49] and sensitivity to Syk and Src inhibitors [11,32] indicate that polymeric PGN uptake occurs primarily, but not exclusively, through Fc or complement receptor (CR) mediated phagocytosis. While similar overall, molecular differences between these two pathways have been reported [2]. The tyrosine kinase Syk has been implicated in both pathways [53,54], and immune responses to PGN are sensitive to Syk inhibitors [11]. In canonical models, FcR phagocytosis promotes actin reorganization through Cdc42 and Rac small GTPases, while CR-phagocytosis utilizes RhoA [55]. Surprisingly, neither Cdc42 inhibition nor RhoA inhibition affected PGN uptake in our study. It is possible that canonical phagocytic models developed with differentiated immune cells might not fully translate to primary naïve human cells used here. Redundant activities for Src family kinases during FcR-mediated phagocytosis have been reported [56] and, likewise, it is possible that other small GTPases, such as Rac, could overcome Cdc42 or RhoA inhibition in our study. As such, molecular details of PGN-triggered phagocytic uptake will require further study.

In general, cells internalize larger particles through actin-mediated uptake, either macropinocytosis or phagocytosis, while smaller cargo can enter through clathrin coated pits, caveolae or similar endocytic structures. The broad size of PGN macromolecules measured in this study, ranging from 100 to 874 nm, indicates that there are no biophysical sizing restrictions that will impede PGN uptake by any of these pathways. Unlike MDP [24] or anthrax toxins [57] that internalize through CME, PGN uptake was clathrin-independent and insensitive to either ES9-17 (shown) or pitstop2 (not shown). We therefore analyzed PGN internalization by clathrin-independent endocytosis (CIE) using a simplified overview detailed by Mayor and Pagano [4]. Accordingly, CIE can be split into dynamin-dependent and independent pathways. The dynamin-dependent CIE can be further divided into caveolin- and/or RhoA-dependent internalizations, while dynamin-independent pathways can be grouped into Cdc42- or Arf6-dependent internalizations. We used an overlapping panel of pharmacologic inhibitors for most of these pathways, except caveolin, for which there is no specific pharmacologic inhibitor reported to date. Our data show that in primary human monocytes, the alternate CIE uptake is dynamin-dependent but independent of RhoA, Cdc42 and Arf6 GTPases.

The identity of the dynamin-dependent CIE is currently elusive, but multiple lines of evidence point to a caveolar uptake of PGN. Caveolin is expressed in myeloid cells [58] and attaches to lipid microdomains enriched in cholesterol, glycosphingolipids and glycosylphosphatidylinositol (GPI)-anchored proteins (GPI-AP), forming flask shaped invaginations named caveolae. Non-opsonic caveolar uptake of bacteria, mediated by GPI-anchored receptors, has been documented and promotes phagolysosomal escape [59]. Similarly, the dynamin-dependent PGN uptake, emphasized after latrunculin inhibition, displays lower lysosomal PGN localization. Interestingly, two putative PGN receptors, CR3 and CD14, can associate with caveolae [58]. Although CR3 could mediate GPI-AP uptake in the absence of serum opsonization, in the presence of serum, it likely supports phagocytic internalization [60]. The caveolar uptake of polymeric PGN seems more likely dependent on CD14, a GPI-anchored protein itself. Although CD14 interaction with polymeric PGN has been documented [43], the mechanisms underlying CD14′s role in cellular responses to PGN are unresolved. In our study, monocyte CD14 staining coalesced in the presence of PGN particles, which could represent aggregation in GPI-AP microdomains, which, in turn, could promote caveolar organization and trafficking. Furthermore, Src phosphorylation of caveolin-1 is needed for caveolae scission [61], and we previously reported reduced PGN responses in the presence of Src inhibitors [11]. It is worth reiterating that Src kinases are also involved in FcR-mediated phagocytosis (see above) and, by themselves, these inhibitors cannot differentiate between these two endocytic pathways. Overall, while the propensity of evidence indicates caveolar PGN uptake as the dynamin-dependent CIE, further studies are needed to define this pathway at the molecular level. Regardless, the actin-insensitive uptake of PGN displayed inefficient phagolysosomal trafficking and, consequently, lowered proinflammatory and procoagulant responses in primary monocyte. Whether or not this pathway is selectively enhanced during bacterial infection resulting in attenuated immune reactions to bacteria and/or peptidoglycan remains to be established.

## 5. Conclusions

In this study, we show that particulate PGN polymers isolated from *Bacillus anthracis* display broad sizes and are internalized through multiple endocytic pathways. Pharmacological screening shows that naïve human monocytes primarily internalize particulate PGN by actin-mediated phagocytosis. To a lesser extent, PGN is internalized by an alternate dynamin-dependent pathway which does not require clathrin, RhoA, Cdc42 or Arf. Actin-mediated internalization supports faster phagolysosomal targeting of PGN than the dynamin pathway and subsequent procoagulant and proinflammatory responses in primary human monocytes.

## Data Availability

All study data used to generate figures are included in the report, except for the summary bar graph representations as detailed in the Materials and Methods Section. Excel datasets used for these figures are available upon request from the corresponding author (narcis-popescu@omrf.org).
